# Electron Transport Layer Optimization for Efficient PTB7:PC_70_BM Bulk-Heterojunction Solar Cells

**DOI:** 10.3390/polym14173610

**Published:** 2022-09-01

**Authors:** Syed Abdul Moiz, Mohammed Saleh Alzahrani, Ahmed N. M. Alahmadi

**Affiliations:** Device Simulation Lab, Department of Electrical Engineering, Umm Al-Qura University, Makkah 21955, Saudi Arabia

**Keywords:** bulk-heterojunction, polymer, solar cell, PEDOT:PSS, PTB7, PCBM, ITO, Zn(O,S), ZnSe, PFN-Br, SCAPS-1D

## Abstract

Bulk-heterojunction (BHJ) polymer solar cells have received a great deal of attention mainly due to the possibility of higher power conversion efficiency for photovoltaic applications. Therefore, in this study, relatively novel polymer BHJ solar cells are proposed (ITO/ETL/PTB7:PC_70_BM/PEDOT:PSS/Au) with various electron transport layers (ETL) such as zinc oxysulfide (Zn(O,S)), zinc selenide (ZnSe), and poly[(9,9-bis(3′-((N,N-dimethyl)-N-ethylammonium)-propyl)-2,7-fluorene)-alt-2,7-(9,9-dioctylfluorene)] dibromide (PFN-Br). Here, each ETL material is selected based on the energy bandgap compatibility with ITO as well as the PTB7:PC_70_BM active layer and is based on other physical properties, which are generally required for efficient photovoltaic responses. Each proposed device is comprehensively optimized and then photovoltaic responses are simulated and compared using the software SCAPS-1D. It was observed that the ITO/Zn(O,S)/PTB7:PC70BM/PEDOT:PSS/Au device offered the highest power-conversion efficiency of up to 17.15% with an open-circuit voltage of 0.85 volts, a short-circuit current of 28.23 mA/cm^2^, and a fill factor of 70.69%.

## 1. Introduction

Over the last few decades, the demand for energy has sharply risen due to economic, social, and industrial growth and development [1,2]. However, the current conventional energy reserves will be abruptly depleted over time. It is unanimously accepted that available energy reserves will not be sufficient to fulfill the enormous energy demand even in the near future [3]. Many researchers believe that the unlimited energy supply from renewable energy resources may be the best solution for fulfilling the never-ending demand for energy [4,5]. Unfortunately, many renewable energy resources are inherently polluting the environment and have some very serious greenhouse requirements. Among the many renewable energy resources, solar energy is considered one of the best options concerning greenhouse technology and has an unlimited and sustainable energy potential that can fulfill the future demand for energy [5,6,7,8].

The best candidate material for solar cells is still silicon, but the costs associated with silicon-processing technology for solar cells are very high due to higher-temperature processing as well as strict clean room technology requirements [9,10]. Therefore, other materials for solar-cell technologies that offer low-cost and highly efficient photovoltaic responses are under intense investigation. Organic/polymer conjugate materials are considered another option, not only for their low cost but also for their highly efficient solar-cell applications [11,12,13], light-emitting diodes [14], flexible transistors [15], and various other types of sensors [16,17]. However, for photovoltaic applications, the reported efficiency of polymer solar cells is still not up to the mark compared to the commercially available silicon-based solar cells.

The working principle of polymer solar cells is very similar to other inorganic solar cells but the photovoltaic processes are much more complicated [18]. Broadly speaking, the photovoltaic processes for polymer solar cells can be classified as the (i) optical absorption of photons, (ii) formation of excitons and diffusion, (iii) dissociation of the excitons into the free carriers; (iv) transportation of the free carriers toward the opposite electrodes; and (v) collection of free holes and electrons at their respective electrodes [19]. When photons strike the surface of active polymer layers, nearly all the photons are absorbed, which generates a huge amount of bound electron–hole pairs as excitons. Generally, these excitons are unfruitfully recombined within 10 to 20 nm of radium due to the strong electrostatic binding energy (exciton binding energy) before reaching their respective transport layers. The remaining fruitful excitons are dissociated into free charge carriers and then transported through the respective transport layers to collect at the electrode and generate some electrical energy for the connected load [20,21]. From the above discussion, it can be inferred that the major obstacle to the efficient polymer solar cell is the lack of exciton dissociation before reaching the respective transport layers. Huge research efforts are being carried out to improve the exciton dissociation for an efficient photovoltaic response.

Among many other solutions, polymer bulk-heterojunction solar cells offer a unique and efficient solution for exciton dissociation and reduction of exciton recombination losses. The polymer BHJ solar cells simply consist of a heterojunction mixture of both electron-donating (polymers) and electron-accepting (fullerenes) materials at the nanoscale domain to enhance the dissociation of excitons, which helps to achieve a reported photovoltaic efficiency in the range of 10–13% [22,23,24]. The bulk heterojunction offers a high interfacial surface area, which plays a vital role in the dissociation of excitons. The selection of the electron-donating and accepting material for efficient BHJ solar cells that combine low bandgap light absorption, adequate energy level positions, and reasonably high carrier mobilities is a challenging task. Recently, a derivative of benzodithiophene generally known as PTB7 has been considered a promising active material for heterojunction and the PTB7:PC_70_BM solar cell shows an excellent photovoltaic response [25,26,27]. Therefore, PTB7:PCBM is selected as the active BHJ layer for this study.

The active PTB7:PCBM layer also interacts with other functional transport materials. These functional materials are generally classified into two well-defined layers: the hole transport and electron transport layers. The main objectives of these functional layers are to selectively transport the electrons/holes as well as block the holes/electrons at the same time [28]. For the hole transport layer, the water-soluble poly(3,4-ethylene dioxythiophene): poly(styrene sulfonate) (PEDOT:PSS) is the most commonly reported material not only for BHJ solar cells [29] but also for polymer-nanowire hybrid solar cells [30], perovskite solar cells [31], and many other types of solar cells and light-emitting diodes [32,33]. PEDOT:PSS has attracted a great deal of research attention due to its excellent and unique properties, for example, it is lightweight, very flexible, and low-cost, has simple processability, is highly transparent, can be deposited on various types of substrates, and has promising electrical and thermoelectric properties [30].

On the other hand, the appropriate materials for the efficient electron transport layer are one of the more serious concerns for polymer-based solar cells and other polymer electronic devices. Because many polymer/non-polymer materials do not fulfill the minimum requirements of an efficient electron transport layer such as (i) a compatible HOMO/LUMO level with the active layer, (ii) acceptable electron mobility, (iii) good environmental and thermal stability, and (iv) simple thin-film processability [34]. Therefore, based on these criteria, zinc oxysulfide (Zn(O,S)), zinc selenide (ZnSe), and poly[(9,9-bis(3′-((N,N-dimethyl)-N-ethylammonium)-propyl)-2,7-fluorene)-alt-2,7-(9,9-dioctylfluorene)] dibromide (PFN-Br) are randomly selected as the electron transport materials for the PTB7:PC_70_BM BHJ active layer, where PEDOT:PSS is used as the hole transport layer.

Similarly, the physics regarding the doping of organic semiconductors either as the hole or electron transport layers for photovoltaic devices is still not very clear, but it has been experimentally observed that the charge transport process for any conducting polymer with a very high doping density is controlled by the ionized impurity scattering, which severely degrades the carrier mobility and hence the overall photovoltaic response [35,36]. Therefore, in this study, all the layer doping densities of the proposed solar cells are optimized up to 10^21^ cm^−3^ for realistic analysis and design.

Simulation and modeling with SCAPS-1D is a very efficient method to systematically investigate the overall photovoltaic response as a function of the various parameters of the electron transport layer. As a result, in this study, we first optimized each layer before investigating and optimizing the photovoltaic response of the ITO/ETL/PTB7:PC70BM/PEDOT:PSS/Au devices as a function of various electron transport layers, such as Zn(O,S), ZnSe, and PFN-Br, and then proposed the most efficient and novel device for photovoltaic applications.

## 2. Device Modeling and Simulation Methods

### 2.1. Simulation Methodology

Various types of software are available for the simulation and modeling of solar cells. Among these, SCAPS-1D is very popular and highly reported due to being open source and reasonable agreements have been observed between experimental findings and SCAPS-1D (SCAPS 3.8, ELIS-University of Gent, Gent, Belgium) simulation results [37,38,39]. Therefore, the modeling of the proposed solar-cell devices was performed with the help of SCAPS-1D software version 3.3.07. The SCAPS-1D offers many optical, electrical, and photovoltaic tools to comprehensively model the overall photovoltaic response of any type of solar cell. Recently, many solar cells including BHJ solar cells were also modeled and reported successfully with the help of SCAPS-1D software [27,40,41,42].

To model the overall photovoltaic response of a solar cell, SCAPS-1D executes four well-defined sets of semiconductor equations such as (i) the Poisson equation (Equation (1)), (ii) continuity equations (Equations (2) and (3)), (iii) charge transport equations (Equations (4)–(6)), and (iv) the absorption coefficient equation (Equation (7)).

(1) $$\frac{d^{2}\emptyset \left(x\right)}{dx^{2}}=\frac{q}{\in_{o}\in_{r}}\;\left(p\left(x\right)-n\left(x\right)+N_{D}-N_{A}+\rho_{p}-\rho_{n}\right)$$

(2) $$\frac{dJ_{n}}{dx}=G-R$$

(3) $$\frac{dJ_{p}}{dx}=G-R$$

(4) $$J=J_{n}+J_{p}$$

(5) $$J_{n}=D_{n}\;\frac{dn}{dx}+\mu_{n\;}n\frac{d\emptyset }{dx}$$

(6) $$J_{p}=-D_{p}\;\frac{dp}{dx}+\mu_{p\;}p\frac{d\emptyset }{dx}$$

(7) $$\alpha \;(\lambda )=\left(A+\frac{B}{h\nu }\right)\;\sqrt{h\nu -E_{g}}$$

where *ϕ* is the electrostatic potential, e is the electric charge, *ε_r_* is the relative permittivity, *ε*_0_ is the absolute permittivity, *N_A_*/*N_D_* are the shallow acceptor/donor impurity densities, *ρ_n_*/*ρ_p_* are the electron/hole density distributions, *n*(*x*)/*p*(*x*) are the electron/hole densities as a function of *x*, *J_p_*/*J_n_* are the hole/electron current densities, *G* is the generation, and *R* is the recombination rate. *D_n_*/*D_p_* are the electron and hole diffusion coefficients, respectively, and *µ_n_*/*µ_p_* are the electron/hole mobilities, respectively. *α*(*λ*) is the absorption coefficient, *h* is the Plank constant, *ν* is the frequency of photons, *E_g_* is the bandgap of the semiconductor absorber layer, and *A*, *B* are arbitrary constants.

### 2.2. Device Structure:

The detailed photovoltaic responses of the proposed devices were investigated, evaluated, and compared as a function of the different electron transport layers. These devices are

1. ITO/Zn(O,S)/PTB7:PC70BM/PEDOT:PSS/Ag (Zn(O,S) device);
2. ITO/ZnSe/PTB7:PC70BM/PEDOT:PSS/Ag (ZnSe device);
3. ITO/PFN-Br/PTB7:PC_70_BM/PEDOT:PSS/Ag (PFN-Br device).

**Figure 1:**
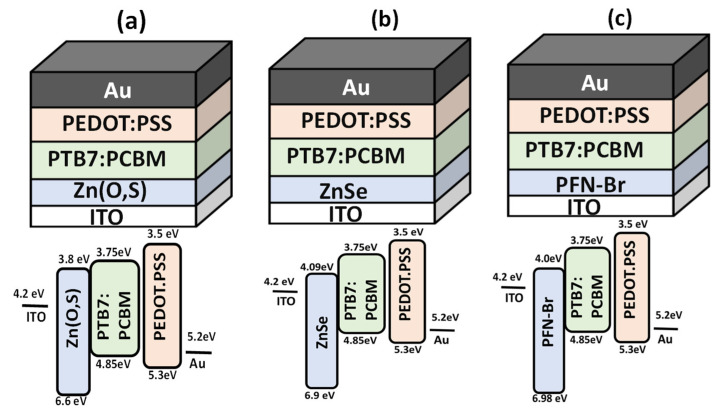
The device block structure and energy band diagram for (**a**) ITO/Zn(O,S)/PTB7:PC_70_BM/PEDOT:PSS/Au, (**b**) ITO/ZnSe/PTB7:PC_70_BM/PEDOT:PSS/Au, (**c**) ITO/PFN-Br/PTB7:PC_70_BM/PEDOT:PSS/Au.

Figure 1 shows the block diagram of each proposed device with its energy bandgap diagram. It can be observed that the conduction band (or LUMO level) of all electron transport layers is in between the conduction band of ITO and PTB7:PCBM, which is the first criterion for the selection of the electron transport layer for any solar cell.

The successive thin-film deposition of a water-soluble conducting polymer even by the spin-coating method is not an issue in modern device fabrication technology. The most popular approach for the fabrication of organic photovoltaic devices involves the use of orthogonal solvents, i.e., the alternating use of organic solvents and water for the application of consecutive layers to prevent the dissolution of the previous layers. Detailed information can be found in the literature [43].

### 2.3. Simulation Parameters

The quality of a simulation depends on the reliability of physical and material parameters. Therefore, all physical and materials parameters required by SCAPS-1D were carefully extracted and cited from the various literature for PTB7:PC_70_BM as the active BHJ layer; PEDOT:PSS as the hole transport layer; and Zn(O,S), ZnSe, and PFN-Br as the electron transport layer, and are listed in Table 1. Similar to many other semiconducting polymers/oxide materials, PTB7, PEDOT:PSS, Zn(O,S), ZnSe, and PFN-Br are considered disordered semiconducting materials. They intrinsically offer both energetic and spatial disorders that can be modeled in terms of traps [44]. The presence of these traps is one of the main causes for observing the poor photovoltaic efficiency of such solar cells. Thus, to improve the quality of the simulation, bulk trap density (10^14^ cm^−3^) is introduced in all electron transport layers as shown in Table 1. Similarly, all photovoltaic simulations were carried out via 100 mW/cm^2^ illumination by a standard solar simulator (A.M. 1.5) under ambient room temperature conditions.

### 2.4. Simulation Flowchart

**Figure 2:**
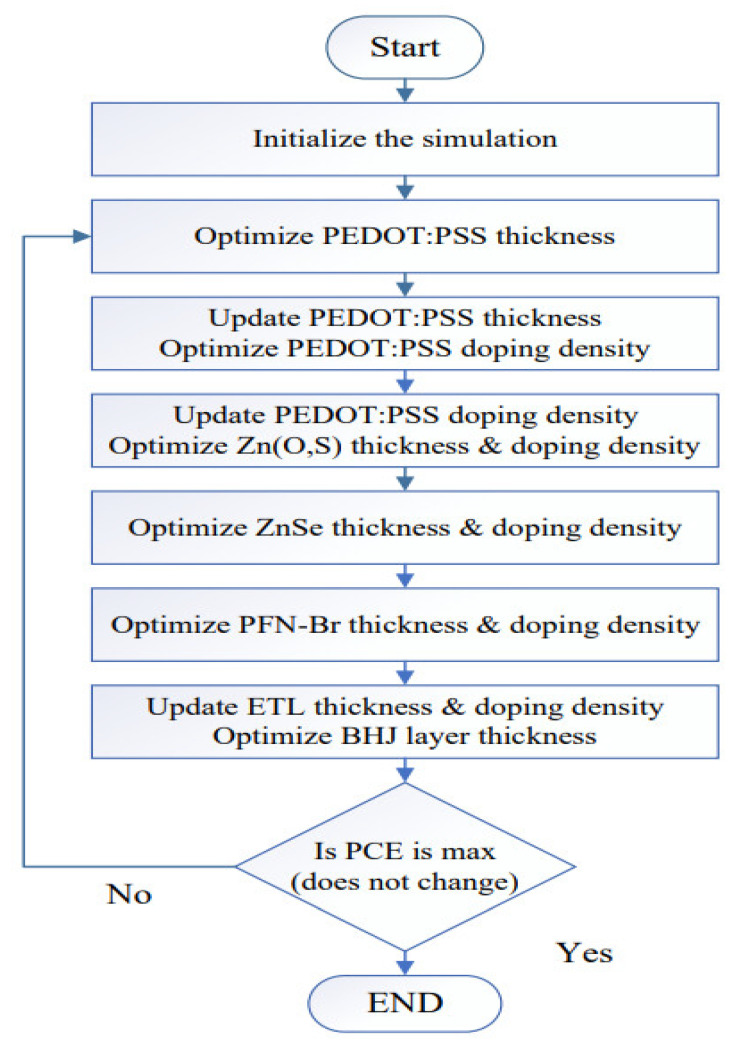
Shows the flow chart used to determine the maximum power-conversion efficiency of the proposed photovoltaic ITO/ETL/PTB7:PC_70_BM/PEDOT:PSS/Au devices with various ETL (Zn(O,S), ZnSe, and PFN-Br) layers.

The general flow chart of the simulation process used in this study to determine the most suitable ETL for the proposed solar cell is shown in Figure 2. The simulation was initialized in the first stage with the suitable boundary conditions and material parameters listed in Table 1. In the second stage, the thickness of PEDOT:PSS as a hole transport layer was optimized and then updated in the SCAPS-1D software before executing the third stage. Similarly, in the third stage, the doping density of PEDOT:PSS was optimized and then updated in the SCAPS software before executing the next stage and so on. In the same way, the ETLs’ (Zn(S,O), ZnSe, and PFN-Br) thickness and doping density were optimized. In the final stage of the simulation, very small, increments/decrements (±5%) of the optimized values were varied to determine which of the proposed devices with the optimized parameters gave the maximum power conversion efficiency for the overall photovoltaic response.

## 3. Results and Discussion

### 3.1. Optimization of PEDOT:PSS Layer Thickness

**Figure 3:**
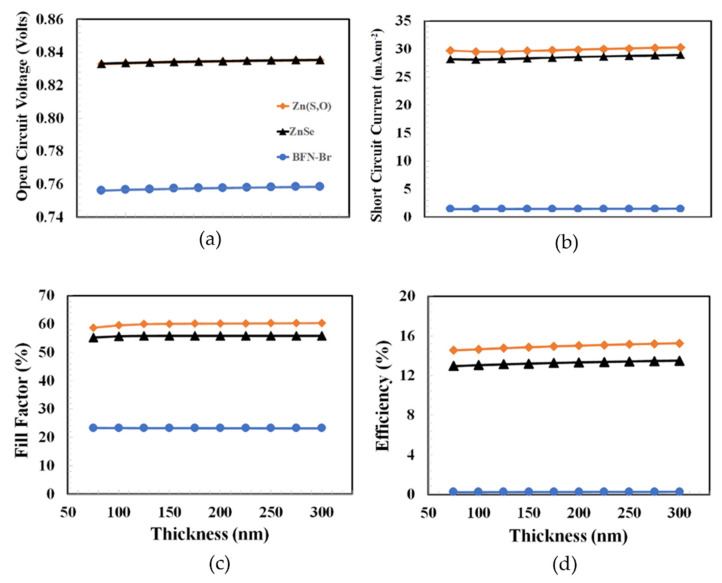
Shows the photovoltaic parameters of (**a**) open-circuit voltage, (**b**) short-circuit current, (**c**) fill factor, and (**d**) power conversion efficiency as a function of PEDOT:PSS thickness for ITO/Zn(O,S)/PTB7:PC_70_BM/PEDOT:PSS/Au, ITO/ZnSe/PTB7:PC_70_BM/PEDOT:PSS/Au, ITO/PFN-Br/PTB7:PC_70_BM/PEDOT:PSS/Au, respectively.

According to the flow chart above, the thickness of PEDOT:PSS was optimized in the first stage, as PEDOT:PSS is the most commonly reported hole transport material for organic/inorganic type solar cells. From the published results, we did not find a PEDOT:PSS thickness above ~300 nm for any efficient photovoltaic device. Therefore, the proposed solar cells were simulated from 75 to 300 nm for the thickness optimization of the hole transport layer. Figure 3 shows the photovoltaic parameters such as open-circuit voltage, short-circuit current, fill factor, and power conversion efficiency as a function of PEDOT:PSS thickness for the Zn(S,O), ZnSe, and PFN-Br devices from 75 to 300 nm. All photovoltaic parameters slightly improved (or were nearly constant) as the thickness of PEDOT:PSS increased. If the maximum power conversion efficiency is the main criteria for determining the optimum thickness of PEDOT:PSS, then it can be inferred from the figure that 300 nm is the optimum thickness of PEDOT:PSS for all the devices, and among these, the device containing an ETL with Zn(O,S) shows the highest power conversion efficiency.

Now, the question arises of how the electron transport layer can affect the thickness of the hole transport layer. The simple answer is that the overall power conversion efficiency depends on the balance of both the electron and hole carriers collected at their respective electrodes [45,46], which in turn directly depends on the nature of the transport layer. Therefore, each device behaves differently as a function of the PEDOT:PSS layer thickness.

### 3.2. Optimization PEDOT:PSS Doping Density

**Figure 4:**
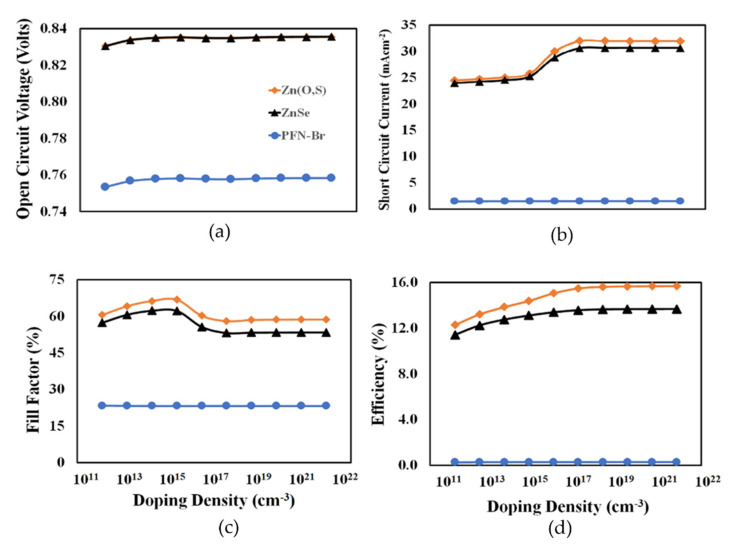
The photovoltaic parameters of (**a**) open-circuit voltage, (**b**) short-circuit current, (**c**) fill factor, and (**d**) power conversion efficiency as a function of the PEDOT:PSS doping density for ITO/Zn(O,S)/PTB7:PC_70_BM/PEDOT:PSS/Au, ITO/ZnSe/PTB7:PC_70_BM/PEDOT:PSS/Au, ITO/PFN-Br/PTB7:PC_70_BM/PEDOT:PSS/Au, respectively.

The doping density of PEDOT:PSS was optimized in the next stage. Figure 4 shows the photovoltaic parameters such as the open-circuit voltage, short-circuit current, fill factor, and power conversion efficiency as a function of the PEDOT:PSS doping density from 10^12^ to 10^21^ cm^−3^ for the Zn(S, O), ZnSe, and PFN-Br devices. Similar to how the thickness of PEDOT:PSS depends on the nature of the electron transport layer as discussed above, the doping density of PEDOT:PSS also depends on the nature and parameters of the electron transport layer. Generally, the proper doping process increases the conductivity and hence improves the overall charge transport process of PEDOT:PSS as a hole transport layer. In Figure 4, the complex photovoltaic responses of the proposed devices can be seen as a function of the doping density, and the nearly constant open-circuit voltage response especially at a higher doping density confirms the formation of the ohmic contact between the anode and BHJ active layer regardless of the doping of the PEDOT:PSS for all the devices [47]. Similarly, the short-circuit current abruptly increased at ~10^16^ cm^−3^ and then became relatively constant, especially for the Zn(O,S) and ZnSe devices. As the maximum power conversion efficiency is the main criteria to determine the optimum doping of PEDOT:PSS, then it can be inferred that efficiency increased up to 10^18^ cm^−3^ for all devices and then became nearly constant so it can be justified that the 10^18^ cm^−3^ is the optimum doping of PEDOT:PSS for each device. Although the Zn(S,O) and ZnSe devices offered relatively good efficiency, the PFN-Br device showed poor photovoltaic performance.

### 3.3. Optimization of Electron Transport Layer Thickness

**Figure 5:**
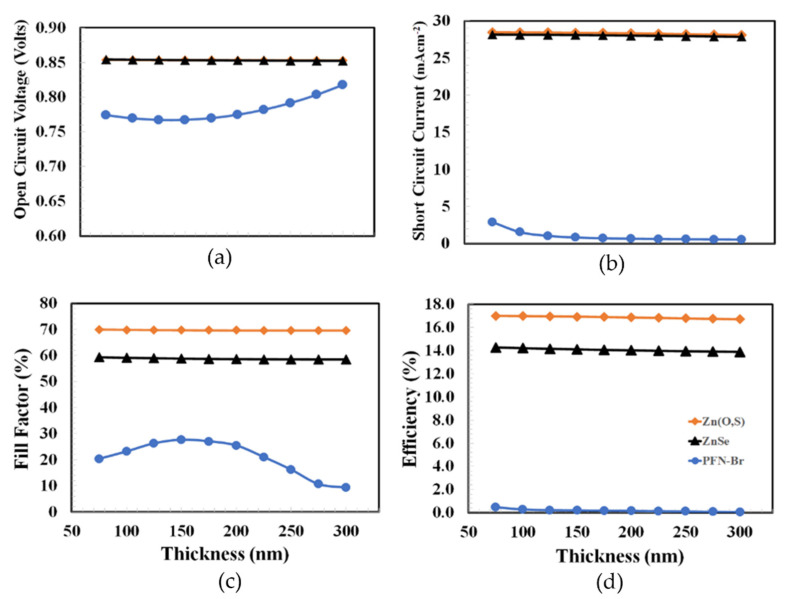
The photovoltaic parameters of (**a**) open-circuit voltage, (**b**) short-circuit current, (**c**) fill factor, and (**d**) power conversion efficiency as a function of electron transport layer (Zn(O,S), ZnSe, PFN-Br) thickness for TO/Zn(O,S)/PTB7:PC_70_BM/PEDOT:PSS/Au, ITO/ZnSe/PTB7:PC_70_BM/PEDOT:PSS/Au, ITO/PFN-Br/PTB7:PC_70_BM/PEDOT:PSS/Au, respectively.

The thickness optimization of the electron transport layer is more crucial compared to PEDOT:PSS because it not only helps to improve the electron transportation and collection process but also offers a path to the photons to manage the solar harvesting in the PTB7:PC_70_BM as the active layer. So, the electron transport layer interacts with the bulk-heterojunction layer on one side and the transparent ITO on the other side. Figure 5 shows the photovoltaic parameters such as open-circuit voltage, short-circuit current, fill factor, and power conversion efficiency as a function of the Zn(S,O), ZnSe, and PFN-Br layers’ thicknesses, which varied from 75 nm to 300 nm. Except for PFN-Br, the electron transport layers (Zn(S,O) and ZnSe) showed very similar photovoltaic responses. For the three devices, the maximum power conversion efficiencies were obtained at 75 nm. Generally, the thin electron transport layer poses some advantages such as higher built-in potential and efficient electron transport. The extra built-in potential plays a significant role in the further dissociation of excitons into the free charge carriers and offers extra drift force to further improve the transport to their respective electrodes [48,49]

### 3.4. Optimization of Electron Transport Layer Doping

In the next step of the simulation, the doping density of the electron transport layer of each device was optimized. Like the hole transport layer, the optical and electrical characteristics of the electron transport layer can also be modified by appropriate doping. The proper doping of the electron transport layer causes a reduction in the bulk resistance of the transport layer, which in turn helps to improve the charge transport as well as the charge collection process and hence the overall photovoltaic performance by the formation of ohmic contact with the transparent ITO electrode [50,51,52].

**Figure 6:**
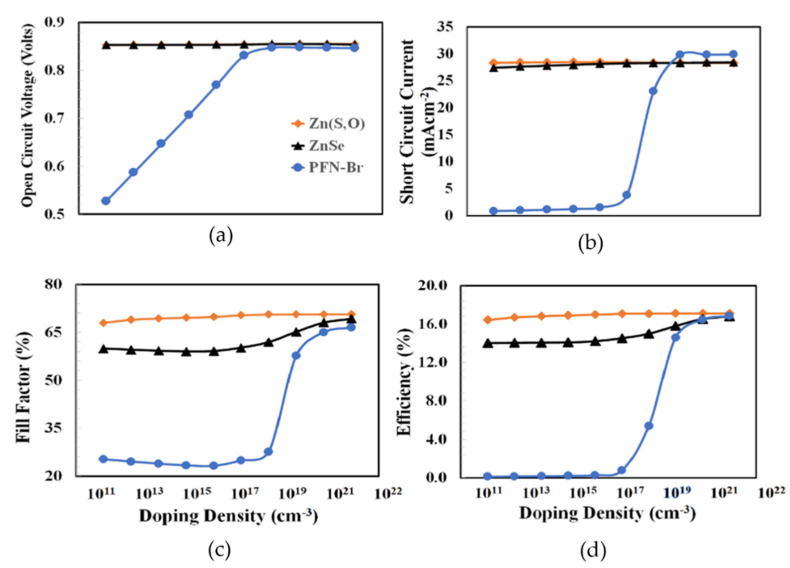
The photovoltaic parameters of (**a**) open-circuit voltage, (**b**) short-circuit current, (**c**) fill factor, and (**d**) power conversion efficiency as a function of the electron transport layer (Zn(O,S), ZnSe, PFN-Br) doping density for ITO/Zn(O,S)/PTB7:PC_70_BM/PEDOT:PSS/Au, ITO/ZnSe/PTB7:PC_70_BM/PEDOT:PSS/Au, ITO/PFN-Br/PTB7:PC_70_BM/PEDOT:PSS/Au, respectively.

Figure 6 shows the photovoltaic parameters such as open-circuit voltage, short-circuit current, fill factor, and power conversion efficiency as a function of the electron transport layer doping density for the Zn(S,O), ZnSe, and PFN-Br devices, respectively. Very complicated photovoltaic responses were observed, where all devices showed improvements in their photovoltaic parameters with the increasing doping density but at different rates. The PFN-Br device showed significant improvements, especially after doping at 10^16^ cm^−3^. Both the ZnSe and Zn(O,S) devices showed very similar increasing responses with the increasing doping density. From the results it can be inferred that the 10^20^ cm^−3^ doping density was the optimum electron transport layer doping density for all the devices, giving the maximum possible power conversion efficiency.

### 3.5. Optimization of BHJ Active Layer Thickness

As an n-i-p-type device structure was used for all the proposed photovoltaic devices, where a PTB7:PC_70_BM bulk-heterojunction layer was used as an insulator layer; therefore, only thickness optimization is required here for the PTB7:PC_70_BM bulk-heterojunction layer. The thickness optimization of an active BHJ layer is a key challenge for the improvement of photovoltaic responses. The optimum thickness of the BHJ layer is the compromise of many factors. On one side, the thickness should be so thin that it allows the dissociation of excitons into free electrons and hole pairs and as well as efficiently transports these free charges toward their respective electrodes [53,54,55]. On the other side, the thickness of the active layer should be thick enough to absorb most of the photons that fall on its surface and generate as many electron–hole pairs as possible. Generally, it is observed from the literature that most of the reported BHJ layer thicknesses for efficient polymer solar cells lie in the range of 80 to 150 nm [56,57,58].

**Figure 7:**
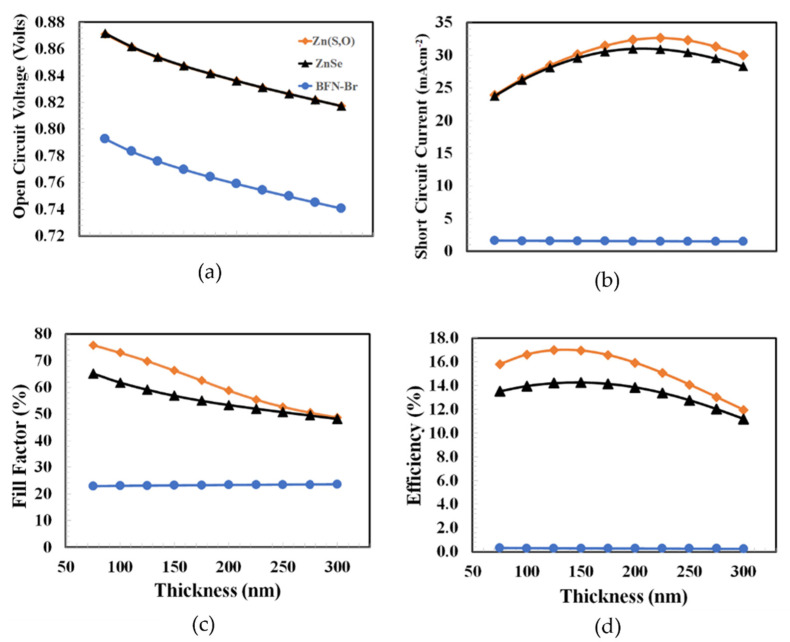
The photovoltaic parameters of (**a**) open-circuit voltage, (**b**) short-circuit current, (**c**) fill factor, and (**d**) power conversion efficiency as a function of the PTB7:PCBM active layer thickness for ITO/Zn(O,S)/PTB7:PCBM/PEDOT:PSS/Au, ITO/ZnSe/PTB7:PCBM/PEDOT:PSS/Au, ITO/PFN-Br/PTB7:PCBM/PEDOT:PSS/Au, respectively.

Figure 7 shows the photovoltaic parameters such as open-circuit voltage, short-circuit current, fill factor, and power conversion efficiency as a function of the active layer thickness for the Zn(S, O), ZnSe, and PFN-B devices, respectively. The figure demonstrates that the open-circuit voltages of all the devices decreased with the increasing BHJ layer thickness because at higher thicknesses, more recombinations take place, which in turn causes a lower open-circuit voltage.

Both short-circuit current and power conversion efficiency behaved with nearly similar responses. However, initially, efficiency increased and reached a maximum value (approximately 125 nm) and then started to decrease. So, it can be inferred from the above discussion that all the parameters discussed above gave the optimum thickness of the BHJ layer at 125 nm to give the maximum power conversion efficiency for the proposed solar cell.

### 3.6. Overall Photovoltaic Response of the Proposed Devices

**Figure 8:**
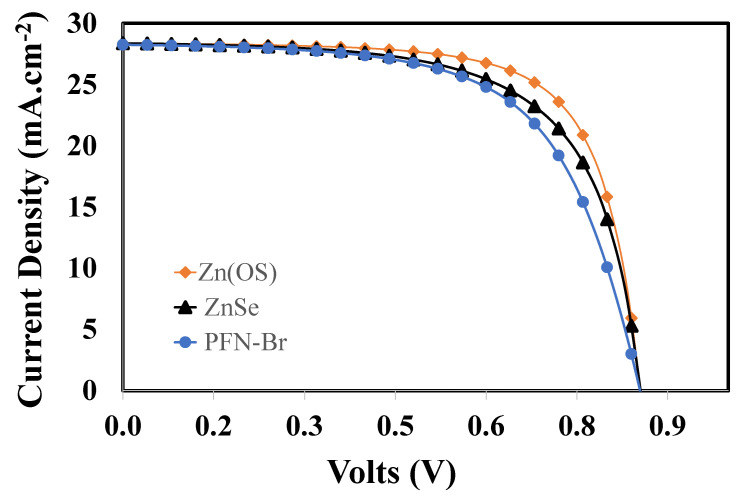
The overall photovoltage parameters of the optimized devices ITO/Zn(O,S)/PTB7:PC_70_BM/PEDOT:PSS/Au, ITO/ZnSe/PTB7:PC_70_BM/PEDOT:PSS/Au, ITO/PFN-Br/PTB7:PC_70_BM/PEDOT:PSS/Au, respectively.

The overall photovoltaic current-voltage response of the proposed ITO/ETL/PTB7:PC_70_BM/PEDOT:PSS/Au devices were simulated and compared as shown in Figure 8, where each layer of each device was already fully optimized concerning film thickness and doping density. Here, Table 2 shows the photovoltaic parameters of each optimized device calculated from the simulated photocurrent responses (Figure 8). The simulation results clearly demonstrate that all devices performed very well, and their maximum power conversion efficiencies were found to be 17.15%, 15.81%, and 15.09% for the Zn(O,S), ZnSe, and PFN-Br devices, respectively.

The photovoltaic parameters such as the open-circuit voltages and short-circuit currents of the devices were found to offer more or less very similar maximum values within a very small range, which demonstrates that the fill factor was the decisive parameter for the selection of the highly efficient photovoltaic Zn(O,S) device compared to the other photovoltaic devices, as shown in Table 2. The fill factor (FF) is a very crucial photovoltaic parameter that demonstrates how the illuminated current-voltage response can be compared with a squared (ideal) current-voltage response for a given organic solar cell. Mathematically, the fill factor can be expressed as

(8) $$FF=\frac{P_{in}}{J_{SC}V_{OC}}=\frac{V_{max}J_{max}}{J_{SC}V_{OC}}$$

where *P_in_*, *V_max_*, *J_max_*, *J_SC_*, and *V_OC_* are the input power, maximum voltage, maximum current, short-circuit current, and open-circuit voltage, respectively. There are several independent parameters, such as mobility, series resistance, shunt resistance, morphology, and molecular weight, of both host and dopants that can significantly affect the fill factor in a very complex way [59,60]. From Table 2, it can be observed that Zn(O,S) offered the highest (70.69%) fill factor compared to ZnSe (65.15%) and PFN-Br (62.49), which could be attributed to the relatively lower built-in potential of Zn(O,S) with the PTB7:PC_70_BM BHJ layer [61]. Such a lower built-in potential not only improves the photovoltaic diode parameters but also improves the electron collection efficiency and hence the fill factor for the efficient photovoltaic responses. So, it can be justified that these parameters were optimized for the ITO/Zn(O,S)/PTB7:PC70BM/PEDOT:PSS/Au device to give the maximum power conversion efficiency of 17.15% compared to the other proposed devices.

## 4. Conclusions

In this study, a comparatively new polymer BHJ active material PTB7:PC_70_BM was sandwiched between efficient hole and electron transport materials. Consequently, in order to determine the most efficient solar cell, we proposed ITO/ETL/PTB7:PC_70_BM/PEDOT:PSS/Au with different electron transport layers, such as zinc oxysulfide (Zn(O,S)), zinc selenide (ZnSe), and poly[(9,9-bis(3′-((N,N-dimethyl)-N-ethylammonium)-propyl)-2,7-fluorene)-alt-2,7-(9,9-dioctylfluorene)] dibromide (PFN-Br), for the photovoltaic devices. Each ETL material was selected based on the energy bandgap compatibility between the ITO and PTB7:PC70BM active layer and other physical parameters. In the first stage, we optimized each layer and then simulated the photovoltaic responses through SCAPS-1D. From the photo current-voltage characteristics, it was observed that all devices behaved with nearly similar responses, whereas the ITO/Zn(O,S)/PTB7:PC_70_BM/PEDOT:PSS/Au device showed a maximum efficiency of up to 17.15% (*V_oc_* = 0.85 V, *J_sc_* = 28.37 mA/cm^2^, and *FF* = 70.69%). It was also observed that these photovoltaic parameters, such as open-circuit voltage and short-circuit current, were the same for all devices, except for the fill factor. It demonstrated that the fill factor was the decisive parameter for maximum efficiency compared to the other proposed devices. The fill factor itself depends on many independent parameters such as mobility, series resistance, shunt resistance, morphology, and the molecular weight of both hosts. Therefore, it can be assumed that all these parameters were optimized for the ITO/Zn(O,S)/PTB7:PC70BM/PEDOT:PSS/Au device to give the highest power conversion efficiency of 17.15% compared to the other proposed devices.

## Figures and Tables

**Table 1:** The physical and material parameters of the active BHJ, hole transport, and electron transport layers used in the simulation, as required by SCAPS-1D.

Parameters	Symbol	Unit	PEDOT:PSS	BHJ	ETL		
					Zn(O,S)	ZnSe	PFN-Br
Thickness	*T*	nm	300	300	75	75	75
Energy Band Gap	*Eg*	eV	1.80	1.10	2.8	2.81	2.98
Electron Affinity	*Χ*	eV	3.50	3.75	3.80	4.09	4.00
Dielectric Permittivity	*ε_r_*	-	3.00	3.90	9.00	8.60	5.00
Effective Density of States at Conduction Band	*N_C_*	cm^−3^	2.2 × 10^18^	6 × 10^19^	2.2 × 10^18^	2.2 × 10^18^	1.0 × 10^19^
Effective Density of States at Valance Band	*N_V_*	cm^−3^	1.8 × 10^19^	2.0 × 10^19^	1.8 × 10^19^	1.8 × 10^18^	1.0 × 10^19^
Electron Thermal Velocity	*V_e_*	cm/s	1.0 × 10^7^	1.0 × 10^7^	1.0 × 10^7^	1.0 × 10^7^	1.0 × 10^7^
Hole Thermal Velocity	*V_h_*	cm/s	1.0 × 10^7^	1.0 × 10^7^	1.0 × 10^7^	1.0 × 10^7^	1.0 × 10^7^
Electron Mobility	*µ_e_*	cm^2^/V·s	1	4.4 × 10^−4^	1.0 × 10^2^	4.0 × 10^2^	2.0 × 10^−6^
Hole Mobility	*µ_h_*	cm^2^/V·s	2.0 × 10^1^	2.5 × 10^−4^	2.5 × 10^1^	1.1 × 10^2^	1.0 × 10^−4^
Donor Density	*N_D_*	cm^−3^	-	1.0 × 10^19^	1.0 × 10^19^	1.0 × 10^19^	1.0 × 10^19^
Hole Density	*N_A_*	cm^−3^	1.0 × 10^18^	1.0 × 10^19^	-	-	-
Defect Density	*N_t_*	cm^−3^	1.0 × 10^14^	1.0 × 10^14^	1.0 × 10^14^	1.0 × 10^14^	1.0 × 10^14^

**Table 2:** The overall photovoltaic parameters for the optimized Zn(O,S), ZnSe, and PFN-Br devices.

ETL	Voc (Volt)	Jsc (mA/cm^2^)	FF (%)	PCE (%)
Zn(O,S)	0.8549	28.376	70.69	**17.15**
ZnSe	0.8551	28.370	65.15	**15.81**
PFN-Br	0.8546	28.255	62.49	**15.09**

## Data Availability

Available on the request.
